# Association between bone mineral density and type 2 diabetes mellitus: a meta-analysis of observational studies

**DOI:** 10.1007/s10654-012-9674-x

**Published:** 2012-03-27

**Authors:** Lili Ma, Ling Oei, Lindi Jiang, Karol Estrada, Huiyong Chen, Zhen Wang, Qiang Yu, Maria Carola Zillikens, Xin Gao, Fernando Rivadeneira

**Affiliations:** 1 Department of Rheumatology, Fudan University, Shanghai, China; 2 Department of Endocrinology, Fudan University, Shanghai, China; 3 Department of Internal Medicine, Erasmus MC, Rotterdam, The Netherlands; 4 Department of Epidemiology, Erasmus MC, Rotterdam, The Netherlands; 5 Genetic Laboratory-Room Ee 579, Department of Internal Medicine, Erasmus MC, PO Box 2040, 3000 CA Rotterdam, The Netherlands

**Keywords:** Bone mineral density, Type 2 diabetes, Meta-analysis

## Abstract

Type 2 diabetes mellitus (T2DM) influences bone metabolism, but the relation of T2DM with bone mineral density (BMD) remains inconsistent across studies. The objective of this study was to perform a meta-analysis and meta-regression of the literature to estimate the difference in BMD (g/cm^2^) between diabetic and non-diabetic populations, and to investigate potential underlying mechanisms. A literature search was performed in PubMed and Ovid extracting data from articles prior to May 2010. Eligible studies were those where the association between T2DM and BMD measured by dual energy X-ray absorptiometry was evaluated using a cross-sectional, cohort or case–control design, including both healthy controls and subjects with T2DM. The analysis was done on 15 observational studies (3,437 diabetics and 19,139 controls). Meta-analysis showed that BMD in diabetics was significantly higher, with pooled mean differences of 0.04 (95% CI: 0.02, 0.05) at the femoral neck, 0.06 (95% CI: 0.04, 0.08) at the hip and 0.06 (95% CI: 0.04, 0.07) at the spine. The differences for forearm BMD were not significantly different between diabetics and non-diabetics. Sex-stratified analyses showed similar results in both genders. Substantial heterogeneity was found to originate from differences in study design and possibly diabetes definition. Also, by applying meta-regression we could establish that younger age, male gender, higher body mass index and higher HbA_1C_ were positively associated with higher BMD levels in diabetic individuals. We conclude that individuals with T2DM from both genders have higher BMD levels, but that multiple factors influence BMD in individuals with T2DM.

## Introduction

Osteoporosis and diabetes are both common human diseases. Albright and Reifenstein [1] reported their coexistence in 1948, but hitherto the association between them remains unclear. Due to the different pathogenesis of type 1 and type 2 diabetes mellitus (T2DM), it is not surprising that there is no uniform entity of diabetic bone disease as such. While decreased bone mineral density (BMD) has consistently been observed in type 1 diabetes mellitus patients [2, 3], studies on BMD investigated in T2DM showed contradictory results with higher, lower or similar values in comparison with healthy control subjects [4–7]. These inconsistent findings may be related to vast differences in study design, BMD measurement technology, differences in site of BMD examination, selection of patients, and presence or absence of complications.

It is well known that advanced age is a risk factor for bone loss and osteoporosis [8, 9]. Some of the attributed mechanisms include increased production of inflammatory cytokines and cellular components, incremental osteoclast precursors generation and decreased bone preservation due to gonadal failure resulting in lower tissue production of sex steroids [10]. Advanced age is also associated with increased fall frequency, lack of exercise, use of drugs that negatively influence bone metabolism and renal function such as drugs prescribed for diabetes and hypertension.

Gender also appears to have an important effect on the relation between BMD and T2DM. Barrett-Connor [11] found that older women with T2DM had higher BMD levels at all sites compared to those with normal glucose tolerance, but this effect was not observed in men. It has also been suggested that obesity and hyperinsulinemia can lead to lower bone turnover in diabetic women [7, 12], so that the adverse effects of estrogen deficiency on bone mass are attenuated and delayed after menopause.

Many studies have shown a difference in population characteristics between type 2 diabetic patients and healthy controls [6, 11, 13, 14]. Diabetic study participants tend to have a higher body mass index (BMI) or weight, increased insulin levels, less physical exercise, higher alcohol consumption and they usually smoke more. The use of diuretics is more common in diabetes. These characteristics might influence bone metabolism independently of diabetes. Paradoxically, an increased risk of osteoporotic fracture in T2DM has been repeatedly demonstrated and this was independent of BMD [13, 15]. This association with fracture adds uncertainty around the actual association between diabetes mellitus and BMD.

The aim of our study was to perform meta-analysis of published articles exploring differences between type 2 diabetics and healthy individuals in BMD levels measured at four anatomical sites. In addition, we evaluated factors influencing BMD variation like sex, age, BMI and glucose control (HbA_1c_ levels) for which a meta-regression was performed to evaluate potential mechanisms by which T2DM influences BMD variation.

## Materials and methods

### Search strategy

A systemic search for all literature that was published in May 2010 or earlier was performed using Pubmed and Ovid online (1950 to present with daily update). The search used MeSH terms “diabetes mellitus” and (“osteoporosis” OR “bone density” or “bone mass”).

### Study selection

Studies were considered eligible for the meta-analysis if (1) they evaluated the association between T2DM and BMD, (2) they were of a cross-sectional, cohort or case–control design, (3) they included healthy subjects without DM as controls, (4) they reported gender-stratified statistics on both individuals with and without T2DM, (5) BMD was measured by dual energy X-ray absorptiometry (DXA) and (6) BMD measurements were expressed as an absolute value in g/cm^2^. In the cases that more than one article presented data from the same study population, the study with more complete reporting of data was selected.

Studies in nonhuman populations, review articles, experimental studies, case reports or studies that lacked controls, studies on type 1 or other types of DM, studies that had no clear definition of T2DM, studies that measured BMD measured by computed tomography, ultrasound or single X-ray absorptiometry were all regarded as ineligible.

Only published results were used and papers in all languages were considered. We supplemented electronic searches by hand-searching reference lists of relevant articles and reviews. The abstracts and titles of primitive collections were initially browsed and all observational studies were extracted. Potentially relevant articles were then considered by double checkout. Disagreements were resolved by discussion between at least two reviewers.

### Data

Quality-scoring varies in meta-analyses of observational studies and no criteria have been internationally accepted to date. Consequently, we appraised each article included in this analysis with the guidelines of the MOOSE group [16]. Some key points were: clear definition of study population, clear and internationally accepted criteria of diagnosing diabetes, description of the coefficient of variation for BMD measurements, consecutive selection of cases, random selection of controls and identification of important confounders. We required that at least 2 studies per site-specific BMD outcome should be available to perform a meta-analysis.

Mean and its standard deviation (SD) of BMD measurements at the calcaneus, femoral neck, total hip, spine and forearm in both diabetics and non-diabetics were extracted to explore the pooled mean difference estimation. If repeated measurements were available in cohort studies we extracted only the measurements at baseline (or the earliest available measurement) as being a cross-sectional study. The mean and standard deviation had to be unadjusted due to large variance of adjusted factors between different studies. If there were statistically significant age differences between patients and controls and the age-adjusted mean and deviation could be found, these data were used; if these were not found the study was excluded. In addition, we performed meta-analysis including the maximally adjusted estimates from studies where available. If sample size of either group in comparison was less than 30, it was not used in our analysis. Gender was considered to be a determinant for subgroup analysis.

If studies lacked SD estimates but provided *P* value, standard error (SE), confidence interval (CI) that related to the mean difference, we estimated SDs using the following methods [17]:

1. From SE to SD: the following formula was used: ${\text{SD}} = \frac{\text{SE}}{{\sqrt {\frac{1}{{{\text{N}}\,{\text{case}}}} + \frac{1}{{{\text{N}}\,{\text{control}}}}} }}$;
2. From CI to SD: SE = (upper limit − lower limit)/3.92 (if 95% CI), then replaced in formula.
3. From *P* value to SD: the corresponding t-value according to *P* value was obtained from a table of the t-distribution with the degrees of freedom given by N_case_ + N_control_ − 2 (where N_case_, N_control_ are the sample sizes); then, assuming ${\text{SE}} = \frac{\text{MD}}{\text{t}}$ (where MD is mean difference between case and control); we finally replaced SE in the formula:

${\text{SD}} = \frac{\text{SE}}{{\sqrt {\frac{1}{{{\text{N}}\,{\text{case}}}} + \frac{1}{{{\text{N}}\,{\text{control}}}}} }}$ (where SD is the average of the SDs of the case and control arms);

### Analyses

The weighted mean difference estimates of BMD in g/cm^2^ comparing diabetes with controls were calculated as DerSimonian and Laird estimators using random effects models. As secondary analyses inverse variance fixed effect models were applied. Publication bias was tested using funnel plots. Tests for heterogeneity were performed by applying the Cochran *Q* test and estimating the degree of inconsistency index (I^2^) [18]. Sources of heterogeneity were investigated by sensitivity analyses stratifying on study design, by excluding studies: on Asian populations, presenting large differences in BMI between cases and controls, and/or having BMD measurements assessed by different densitometers. All analyses were conducted with the use of Review Manager, version 5.0 (Revman, The Cochrane Collaboration; Oxford, UK) and Comprehensive Meta-analysis version 2 (Biostat, Inc., Englewood, USA). To estimate the effects of gender, age, BMI and HbA_1C_ on the BMD measured at the different sites a meta-regression analysis was performed using STATA 11.0 (StataCorp LP, USA).

## Results

**Fig. 1:**
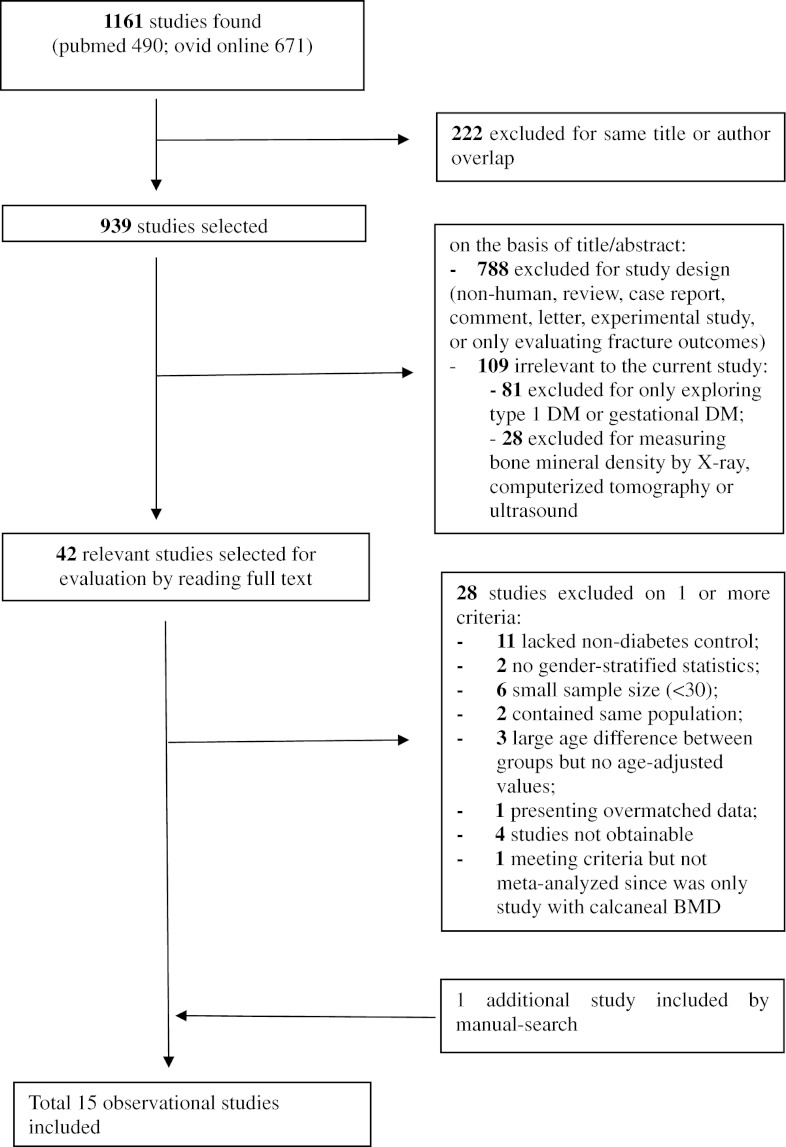
Flow diagram of the study-selection process. *DM* diabetes mellitus, *CT* computed tomography, *US* ultrasound

Figure 1 shows a flow diagram describing the study selection process. The initial search yielded 1,161 research reports, of which 222 were excluded for having the same title or authors; 788 were excluded due to not eligible study design (including non-human studies, review articles, case reports, comment, letter, experimental study, and/or fracture-only outcome). Additional 109 studies were found irrelevant to the original research question and excluded because the disease of interest was either type 1 or gestational DM (81 studies); or for not measuring bone mass using DXA, i.e. by single X-ray absorptiometry, CT or ultrasound (28 studies). Of the 42 remaining studies, 11 either lacked non-diabetic controls at all or did not report means and standard deviations in non-diabetic controls [19–29]. In addition, six studies had small sample sizes (n < 30) in either group of comparison [30–35]. The study population of two studies was used in follow-up reports [4, 36]. In three studies there was a big age difference between individuals with diabetes and those without diabetes, but the investigators did not adjust for it [37–39]. One study matched cases and controls by age and BMI and presented data only on post-matching [40]. The original articles of four articles could not be retrieved [41–44]. All of these aforementioned studies were excluded. One study cited as reference in one of the research reports was traced and satisfied the inclusion criteria [45]. In one research report the results of gender-specific BMD analyses was mentioned, but not listed in detail [14]. We contacted the researchers and were able to retrieve this information. The study of Perez et al. [46] found a significantly increased calcaneal BMD in female but not in males subjects with diabetes. No meta-analysis was attempted for this site since this was the only study that evaluated BMD at the calcaneus. Since no SD’s for male comparison groups could be retrieved for the paper by Barrett-Connor et al. we were not able to include these results for men. As we extracted only a single measure and didn’t examine repeated measurements, cohort studies were analyzed as cross-sectional using the baseline or earliest available measurement. A total of 15 observational studies (9 case–control, 6 cross-sectional) were included in our meta-analysis (3,437 diabetics and 19,139 controls) [5–7, 11, 12, 14, 45, 47–54]. Table 1 indicates the quality evaluation of all studies. We did not observe indication of publication bias on the Funnel Plots (data not shown), with the effect magnitude of larger studies being closer to and smaller studies largely equally distributed at both sides of the summary estimate.

**Table 1:** Aspects of quality and design of the included articles

Reference	Study design	Clear definition of study population	Clear criteria of diagnosing diabetes	Precise control (CV) for BMD measurement	Consecutive selection of cases	Random selection of controls	Identification of important confounders
Barrett-Connor [11]	Cross-sectional	Yes	WHO criteria	NA	Yes	Yes	Yes
Sosa [47]	Case–control	Yes	NDDG criteria (Canada)	Yes	No	No (age-matched)	Yes
Tuominen [48]	Case–control	Yes	NA (hospital database)	NA	Yes	Yes	Yes
Kao [6]	Cross-sectional	Yes	WHO criteria, self-reported	Yes	Yes	Yes	Yes
Dennison [49]	Cross-sectional	Yes	OGTT	Yes	Yes	Yes	Yes
Bridges [50]	Case–control	Yes	NA (hospital database)	NA	No	Yes	Yes
Gerdhem [12]	Cross-sectional	Yes	Self-reported	Yes	Yes	Yes	Yes
de Liefde [14]	Cross-sectional	Yes	Screening (OGTT), drug use	Yes	Yes	Yes	Yes
Majima [7]	Case–control	Yes	OGTT	NA	Yes	Yes	Yes
Schwartz [51]	Case–control	Yes	FPG, OGTT, self-reported	Yes	Yes	Yes	Yes
Bonds [45]	Cross-sectional	Yes	Self-reported, drug use	NA	Yes	Yes	No
Rakic [52]	Case–control	Yes	WHO criteria	Yes	Yes	No (age-, sex-matched)	Yes
Hadzibegovic [53]	Case–control	Yes	NA	NA	Yes	Yes	Yes
Anaforoglu [54]	Case–control	Yes	NA (hospital database)	NA	Yes	No (age-matched)	Yes
Yaturu et al. [5]	Case–control	NA	NA	Yes	Yes	Yes	Yes

Table 2 shows study population characteristics and the reported effect of covariates on the association between BMD and T2DM. Out of five studies performed in the US, one had included Mexican–American women [6] and one had white and black participants [51]. One study was done in Eastern Asia [7] and another two in Eastern Europe [53, 54]. The remaining eight studies collected data in Western Europe and Oceania. Participants in all study populations were aged 25 years and over and approximately 70 % were middle-aged or older. In addition, Table 2 shows that the most common covariates considered by the studies were BMI or weight, cigarette smoking, alcohol use, physical activity, diuretic use, calcium intake, estrogen use (women), menopause status (women), age at menarche (women), insulin level, HbA_1C_ and alkaline phosphatase. Table 3 shows the population characteristics of the source studies by gender.

**Table 2:** Characteristics of the study population and the effects of covariates on BMD

Reference	Ethnicity/nation	Gender (%women)	Age (y)	Covariates: comparison diabetes and non-diabetes (*P* value)	Findings
Barrett-Connor [11]	USA	61	55–88	NS: BMI, cigarette smoking, alcohol use (men), regular exercise, diuretic use (women), estrogen use <0.01: alcohol use (women), diuretic use (men)	No change of statistical significance of mean difference when adjusted for covariates
Sosa [47]	Spain	100	61.3/58.8	<0.05: weight	Analysis of variance (ANOVA) was used to examine the effects of diabetes and weight in bone mass. There were no statistical differences.
Tuominen [48]	Finland	52	45–64	NS: BMI <0.01: use of loop diuretics	No change of statistical significance of mean difference when adjusted for covariates
Kao [6]	USA	64	30–96	NS: diuretics (women), smoking (men), physical activity, calcium intake, estrogen use, menopause status <0.05: diuretics (men), smoking (women), alcohol, BMI	After adjusted for covariates, the increase of BMD attenuated but the decrease expanded No significant difference between newly diagnosed and previously diagnosed diabetes Positive correlation (hip, forearm): insulin level
Dennison [49]	UK	45	59–72	NA	After adjustment for BMI, all relationship were diminished, even femoral neck and total femur lose significance
Bridges [50]	UK	0	≥25	<0.01: BMI	Positive correlation: BMI No significant correlation: HbA_1C_, disease duration, diabetic complication
Gerdhem [12]	Sweden	100	75	<0.001: body weight	Adjustment for body weight, significance remained but the mean difference attenuated
de Liefde [14]	Netherlands	61	≥55	<0.05: BMI, lower limb disability, smoking, baseline use of thiazides, baseline use of loop diuretics	No change of statistical significance of mean difference when adjusted covariates
Majima [7]	Japan	56	≥32	NS: BMI, Scr <0.01: FPG	Positive correlation: BMI, insulin level, HbA_1C_ No significant correlation: FPG
Schwartz [51]	USA	50	70–79	NS: IL-6 (black, white men), current smoker, walking speed (black), statin use, oral estrogen use, renal insufficiency(black), vitamin D supplement use <0.05: weight, weight change, IL-6 (white women), walking speed (white), renal insufficiency (white)	After adjusting for covariates, white women with DM lost more BMD per year on average than those without DM Adjustment for weight loss resulted in the largest attenuation in the association between DM and bone loss
Bonds [45]	USA	100	64.9/63.5	NA	NA
Rakic [52]	Australia	44	Female: 65.5/64.8 Male: 66.0/66.3	NA	Adjustment for BMI, statistical significance of the mean differences was lost at the spine (women) and hip (men) Negative correlation: serum triglycerides, HbA_1C_
Hadzibegovic [53]	Croatia	100	41–84	NS: BMI, menarche age, alkaline phosphatase	Positive correlation: BMI, menarche age Negative correlation: alkaline phosphatase
Anaforoglu [54]	Turkey	100	61.9/60.1	<0.05: BMI, calcium intake	Adjustment for BMI and calcium intake, no statistical significant change
Yaturu et al. [5]	USA	0	67.5/66.2	<0.05: BMI, smoking, alcohol	Matched covariates, statistical significance of mean difference at the spine was lost and at the hip was cut down

*BMI* body mass index, *NS* not significant, *NA* no data, *Scr* serum creatinine, *FBG* fasting blood glucose

**Table 3:** Population characteristics of the source studies by gender

Study	Female					Male				
	Age (years)	BMI (kg/m^2^)	HbA_1c_ (%)	Serum creatine (μmol/L)	Disease duration (years)	Age (years)	BMI (kg/m^2^)	HbA_1c_ (%)	Serum creatine (μmol/L)	Disease duration (years)
Barrett-Connor [11]	76.0	26.3	6.7	99.7	NA	76.0	26.3	6.7	99.7	NA
Tuominen [48]	63.3	25.3	9.8	NA	NA	63.3	25.3	9.8	NA	NA
Kao [6]	54.3	33.0	NA	NA	NA	54.3	33.0	NA	NA	NA
Dennison [49]	64.8	26.6	NA	NA	NA	64.8	26.6	NA	NA	NA
Bridges [50]	62.8	31.4	8.9	NA	10.1	62.8	31.4	8.9	NA	10.1
de Liefde [14]	69.6	25.8	NA	96.2	NA	69.6	25.8	NA	96.2	NA
Majima [7]	62.8	23.6	7.8	66.3	NA	62.8	23.6	7.8	66.3	NA
Schwartz [51] (white)	73.7	NA	7.2	NA	7.4	73.7	NA	7.2	NA	7.4
Schwartz [51] (black)	74.0	NA	8.2	NA	9.5	74.0	NA	8.2	NA	9.5
Rakic [52]	66.0	29.0	7.4	94.0	8.7	66.0	29.0	7.4	94.0	8.7
Yaturu et al. [5]	67.5	30.1	NA	106.1	NA	67.5	30.1	NA	106.1	NA

Table 4 presents BMD levels in diabetics and non-diabetics at four skeletal sites across the different studies, also including subgroup analysis by gender. At the femoral neck, all studies except for Yaturu et al. [5] and Majima [7] found a higher BMD in subjects with diabetes. At the total hip, all referred studies showed significantly higher BMD in diabetics. At the lumbar spine, almost all of the studies reported a higher BMD in diabetics. These differences were statistically significant in the vast majority. At the forearm there were no significant differences between diabetics and non-diabetics in all analyses. No major differences between genders were found.

**Table 4:** Unadjusted/age-adjusted, gender-specific BMD in patients with diabetes and controls per skeletal site (mean ± SD g/cm^2^)

Reference	Female				Male			
	Sample size (case/control)	Diabetes	Non-diabetes	*P* value	Sample size (case/control)	Diabetes	Non-diabetes	*P* value
Skeletal site of BMD measurement: femoral neck								
Barrett-Connor [11]	37/237	0.664 ± 0.118^a^	0.610 ± 0.118^a^	<0.01	41/139	0.747 ± NA	0.744 ± NA^a^	NS
Sosa [47]	47/252	0.756 ± 0.146	0.737 ± 0.115	NS				
Tuominen [48]					34/240	0.881 ± 0.143	0.872 ± 0.131	NS
Dennison [49]	32/278	0.830 ± 0.120	0.740 ± 0.110	<0.0001	33/349	0.900 ± 0.130	0.840 ± 0.110	0.03
Gerdhem [12]	67/961	0.820 ± 0.120	0.740 ± 0.110	<0.0001				
de Liefde [14]	326/3,049	0.859 ± 0.148	0.826 ± 0.134	<0.0001	254/2,195	0.946 ± 0.149	0.914 ± 0.136	0.0003
Majima [7]	81/54	0.620 ± 0.153	0.660 ± 0.118	NS	64/41	0.759 ± 0.137	0.767 ± 0.108	NS
Schwartz [51] (white)	97/383	0.670 ± 0.110	0.640 ± 0.100	<0.05	153/395	0.800 ± 0.120	0.760 ± 0.130	<0.05
Schwartz [51] (black)	125/225	0.790 ± 0.130	0.730 ± 0.130	<0.05	105/169	0.890 ± 0.140	0.830 ± 0.120	<0.05
Rakic [52]	86/86	0.808 ± 0.153	0.722 ± 0.103	<0.001	108/108	0.851 ± 0.128	0.802 ± 0.129	0.01
Hadzibegovic [53]	130/166	0.870 ± 0.132	0.832 ± 0.134	<0.05				
Anaforoglu [54]	206/61	0.770 ± 0.110	0.730 ± 0.120	0.280				
Yaturu et al. [5]					735/3,458	0.892 ± 0.244^b^	0.930 ± 0.176^b^	<0.0001
Skeletal site of BMD measurement: total hip								
Schwartz [51] (white)	97/383	0.790 ± 0.120	0.750 ± 0.120	<0.05	153/395	0.950 ± 0.130	0.930 ± 0.140	<0.05
Schwartz [51] (black)	125/225	0.910 ± 0.150	0.840 ± 0.150	<0.05	105/169	1.070 ± 0.150	1.000 ± 0.130	<0.05
Bonds [45]	469/5,916	0.900 ± 0.160	0.840 ± 0.140	<0.01				
Rakic [52]	86/86	0.993 ± 0.173	0.848 ± 0.118	<0.001	108/108	1.060 ± 0.156	1.013 ± 0.158	0.038
Skeletal site of BMD measurement: spine								
Barrett-Connor [11]	37/237	0.962 ± 0.225^a^	0.859 ± 0.225^a^	<0.01	41/136	1.081 ± NA^a^	1.069 ± NA^a^	NS
Sosa [47]	47/252	0.898 ± 0.137	0.892 ± 0.138	NS				
Kao [6]	98/285	1.071 ± 0.188^b^	1.011 ± 0.236^b^	<0.01	55/162	1.057 ± 0.222^b^	1.063 ± 0.255^b^	NS
Dennison [49]	32/278	1.070 ± 0.180	0.940 ± 0.180	0.0001	33/349	1.160 ± 0.120	1.070 ± 0.160	0.005
Gerdhem [12]	67/961	1.070 ± 0.230	0.990 ± 0.190	0.0001				
de Liefde [14]	327/3,052	1.084 ± 0.188	1.030 ± 0.179	<0.0001	255/2,205	1.196 ± 0.209	1.161 ± 0.196	0.007
Majima [7]	81/54	0.861 ± 0.193	0.831 ± 0.162	NS	64/41	0.972 ± 0.176	0.975 ± 0.108	NS
Bonds [45]	472/5,922	1.040 ± 0.190	0.970 ± 0.170	<0.01				
Rakic [52]	86/86	1.031 ± 0.171	0.948 ± 0.152	<0.001	108/108	1.117 ± 0.176	1.102 ± 0.191	0.55
Hadzibegovic [53]	130/166	0.903 ± 0.165	0.824 ± 0.199	<0.001				
Anaforoglu [54]	206/61	0.900 ± 0.160	0.870 ± 0.150	0.264				
Yaturu et al. [5]					735/3,458	1.223 ± 0.217^b^	1.149 ± 0.176^b^	<0.0001
Skeletal site of BMD measurement: forearm								
Kao [6]	98/285	0.477 ± 0.079^b^	0.463 ± 0.101^b^	NS	55/162	0.535 ± 0.096^b^	0.547 ± 0.102^b^	NS
Bridges [50]					90/50	0.560 ± 0.097^c^	0.560 ± 0.090^c^	NS
Majima [7]	81/54	0.493 ± 0.109	0.547 ± 0.095	<0.01	64/41	0.665 ± 0.092	0.721 ± 0.080	<0.05
Rakic [52]	86/86	0.540 ± 0.066	0.481 ± 0.068	<0.001	108/108	0.641 ± 0.062	0.627 ± 0.063	0.09
Hadzibegovic [53]	130/166	0.496 ± 0.065	0.485 ± 0.081	NS				
Anaforoglu [54]	206/61	0.48 ± 0.050	0.49 ± 0.010	0.696				

SD written as NA if neither exact *P* value, SE or CI was available

^a^Using the formula from *P* value to SD

^b^Using the formula from SE to SD

^c^Using the formula from CI to SD

Some reports concluded that the association remained significant despite the fact that the effect size decreased remarkably after correcting for aforementioned covariates [6, 11, 12, 14, 48, 54]. In others, the association disappeared or even shifted in the opposite direction after adjustment for covariates, particularly in the case of BMI or weight [5, 49, 51, 52]. We performed meta-analysis for maximally adjusted estimates where available, which did not significantly alter previously calculated mean differences. Nearly all studies found that BMI was positively correlated with BMD. There was some evidence suggesting that other factors such as insulin levels also had a positive correlation with BMD [7]. In contrast, HbA_1c_ levels had positive [7], negative [51] or no correlation [50] with BMD. In a follow-up study, Schwartz [51] found that after adjustment for covariates white women with T2DM lost on average more BMD per year than those without DM.

**Fig. 2:**
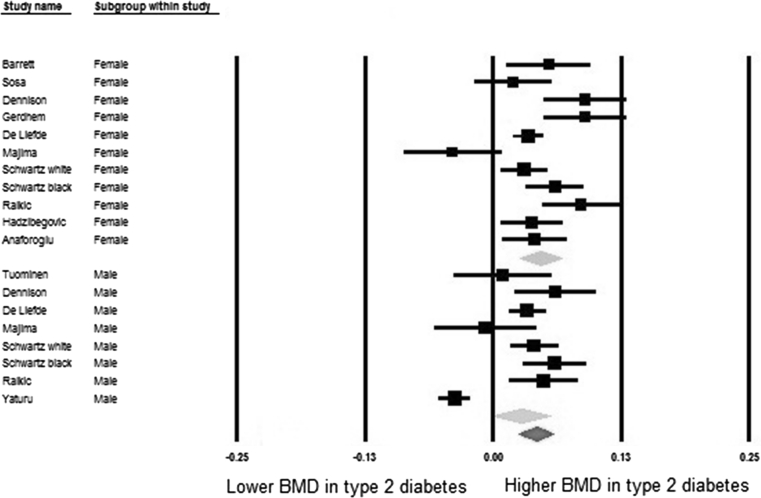
Forest plot for mean femoral neck bone mineral density. Difference in means (g/cm^2^) and 95% confidence interval for femoral neck bone mineral density between comparison groups with and without Type 2 Diabetes Mellitus, stratified per study and gender. *Diamonds* represent joint estimate for subgroups of available studies for women (*upper*) and men (*middle*), respectively. Pooled estimate for all studies displayed with the *diamond* at the *bottom*

**Fig. 3:**
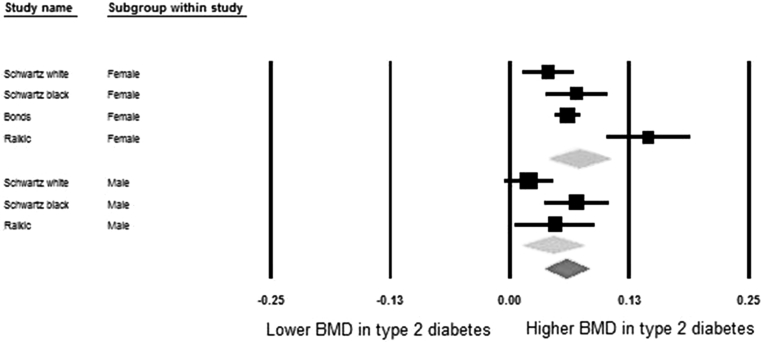
Forest plot for mean hip bone mineral density. Difference in means (g/cm^2^) and 95% confidence interval for hip bone mineral density between comparison groups with and without type 2 diabetes mellitus, stratified per study and gender. *Diamonds* represent joint estimate for subgroups of available studies for women (*upper*) and men (*middle*), respectively. Pooled estimate for all studies displayed with the *diamond* at the *bottom*

**Fig. 4:**
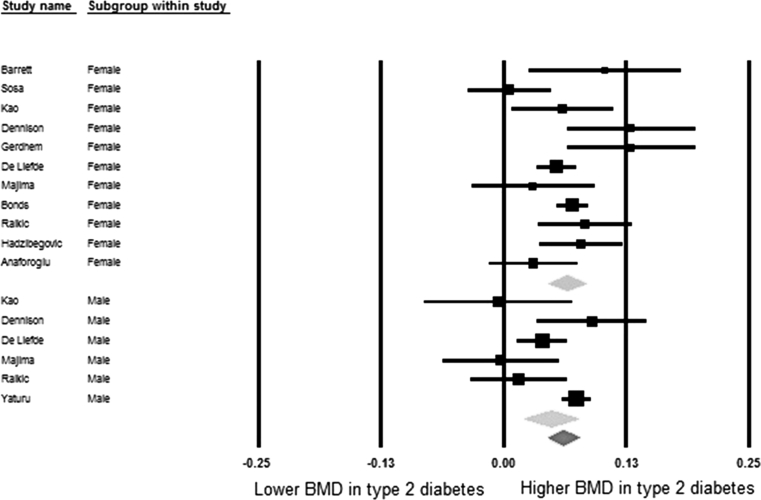
Forest plot for mean spine bone mineral density. Difference in means (g/cm^2^) and 95% confidence interval for spine bone mineral density between comparison groups with and without type 2 diabetes mellitus, stratified per study and gender. *Diamonds* represent joint estimate for subgroups of available studies for women (*upper*) and men (*middle*), respectively. Pooled estimate for all studies displayed with the *diamond* at the *bottom*

**Fig. 5:**
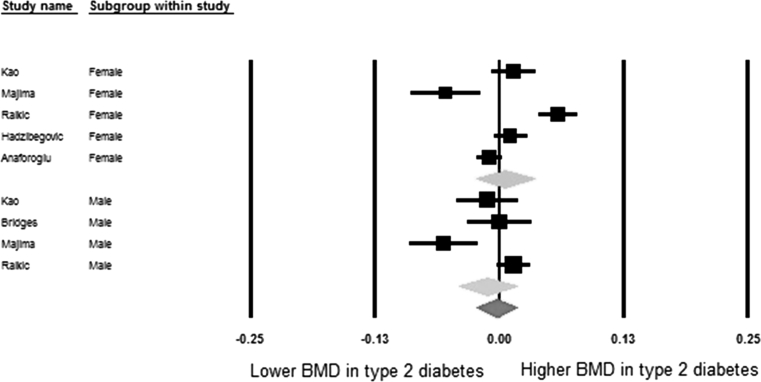
Forest plot for mean forearm bone mineral density. Difference in means (g/cm^2^) and 95% confidence interval for forearm bone mineral density between comparison groups with and without type 2 diabetes mellitus, stratified per study and gender. *Diamonds* represent joint estimate for subgroups of available studies for women (*upper*) and men (*middle*), respectively. Pooled estimate for all studies displayed with the *diamond* at the *bottom*

Table 5 shows meta-analysis results of pooled mean differences and corresponding 95% confidence intervals of BMD values between diabetic and non-diabetic individuals. In the pooled meta-analyses the differences were 0.04 (95% CI: 0.02, 0.05) at the femoral neck, 0.06 (95% CI: 0.04, 0.08) at the hip, 0.06 (95% CI: 0.04, 0.07) at the spine, and −0.003 (95% CI: −0.02, 0.02) at the forearm, respectively. In the sex-stratified analysis these differences were most pronounced for females, being 0.04 (95% CI: 0.03, 0.06), 0.07 (95% CI: 0.04, 0.11), 0.07 (95% CI: 0.05, 0.09), 0.01 (95% CI: −0.02, 0.03) at the femoral neck, hip, spine, and forearm, respectively. In males these differences were statistically significant at the hip 0.04 (95% CI: 0.01, 0.08) and spine 0.05 (95% CI: 0.02, 0.07). The meta-analysis result in males was non-significant at the femoral neck 0.03 (95% CI: 0.00, 0.05) and forearm −0.01 (95% CI: −0.04, 0.02). This information is displayed in more detail in the forest plots of Figs. 2, 3, 4, and 5.

**Table 5:** Pooled mean differences of BMD comparing diabetes with non-diabetes

Site	Groups	Number of studies	Sample size (case/control)	Mean difference of BMD (g/cm^2^)	*P* value	Heterogeneity	
						I^2^ (%)	*Q* test *P* value
Femoral neck	Total	12	2,720/12,707	0.04 [0.02, 0.05]	<0.00001	83	<0.0001
	Female	10	1,234/5,752	0.04 [0.03, 0.06]	<0.00001	71	0.0002
	Male	7	1,486/6,955	0.03 [0.00, 0.05]	0.09	87	<0.0001
Hip	Total	3	1,143/7,282	0.06 [0.04, 0.08]	<0.00001	78	0.0002
	Female	3	777/6,610	0.07 [0.04, 0.11]	<0.00001	82	0.001
	Male	2	366/672	0.04 [0.01, 0.08]	0.007	63	0.07
Spine	Total	12	2,833/17,677	0.06 [0.04, 0.07]	<0.00001	66	<0.0001
	Female	11	1,583/11,354	0.07 [0.05, 0.09]	<0.00001	62	0.003
	Male	6	1,250/6,323	0.05 [0.01, 0.07]	0.008	74	0.002
Forearm	Total	6	918/1,013	−0.003 [−0.02, 0.02]	0.90	88	<0.0001
	Female	5	601/652	0.01 [−0.02, 0.03]	0.68	93	<0.0001
	Male	4	317/361	−0.01 [−0.04, 0.02]	0.44	79	0.003

The weighted mean difference estimates of BMD were calculated as DerSimonian and Laird estimators using random effects models

Tests for heterogeneity were performed by applying the Cochran *Q* test

The heterogeneity (*Q*) tests showed significant differences between individual studies (*P* < 0.01) at all sites in the total group and sex-specific analyses (Table 5). Still, point estimates and statistical significance from fixed effects models were very similar to those derived from random effects models. We further performed sensitivity analyses to identify potential sources of the observed heterogeneity. Subgroup analyses per study design (case–control/cross-sectional) showed that case–control studies had effect estimates with larger variation around the pooled estimate thereby increasing the heterogeneity. For the femoral neck BMD analysis the largest source of heterogeneity was traced back to one study by Yaturu et al. [5]. This study include only men and observed a positive relation with lumbar spine and a negative one for femoral neck; after removing this study the I^2^ statistic dropped from 81 to 57 %. Another study in Asians also displayed estimates in the opposite direction for different outcomes though not significant [7]. Removing seven studies with significantly different BMI between diabetes and non-diabetes [5, 12, 14, 47, 50, 51, 54] or six studies that did not use a densitometer manufactured by Hologic incorporation (USA) [5, 12, 14, 48, 50] from the analyses showed no significant influence on the observed heterogeneity, except for the femoral neck BMD analysis, but this was largely attributable to the large heterogeneity brought in by the Yaturu et al. study [5].

The results of a meta-regression on BMD by sex, age, BMI and glucose control (HbA_1c_ levels) is presented in Table 6 for individuals from the diabetic group of the studies. Being a woman was associated with significantly lower BMD levels at all four anatomical sites, as compared to men. Age was negatively associated with BMD at hip but positively at the lumbar spine. Higher BMI was a strong determinant of higher BMD at the femoral neck and lumbar spine, with no apparent effect on forearm BMD. Higher HbA_1C_ levels (reflecting lesser glucose control) resulted in higher BMD at the femoral neck and total hip.

**Table 6:** Meta-regression results for BMD for individuals from the diabetic group of the studies

Site	Gender (female–male)	Age (years)	BMI (kg/m^2^)	HbA_1c_ (%)
Femoral neck	−0.114 ± 0.012*	0.002 ± 0.002	0.022 ± 0.002*	0.045 ± 0.013*
Hip	−0.119 ± 0.021*	−0.015 ± 0.003*	–	0.117 ± 0.024*
Spine	−0.164 ± 0.018*	0.030 ± 0.006*	0.029 ± 0.004*	0.241 ± 0.090*
Forearm	−0.150 ± 0.050*	0.001 ± 0.013	−0.001 ± 0.006	−0.062 ± 0.052

Values are regression coefficients ± SEM, * *P* value < 0.05

## Discussion

Our study provides insights into the inconsistently reported relationship between T2DM and BMD. In line with what is suggested by the majority of reviewed studies our meta-analysis concluded that overall individuals with T2DM have about 25–50 % SD higher BMD compared to non-diabetic control subjects.

In this study we found no strong evidence for skeletal site specificity of this association. Subjects with T2DM had elevated BMD at the femoral neck, hip, and spine. No major differences in BMD at the forearm were seen but there are no obvious biological reasons we can attribute to them. This lack of association with forearm BMD may be the consequence of limited sample size. We also found no strong evidence suggesting there is sex-specificity in the observed BMD differences between diabetics and non-diabetics. BMD differences seem larger in women than in men but power limitations can also play a role. We did find considerable heterogeneity influencing the association as reflected by a high I^2^ statistic. This large heterogeneity could most probably stem from a large variation in types of study design, diagnostic definitions and individual characteristics that were not considered by each study. We did sensitivity analyses trying to find sources of heterogeneity and concluded that study design and Asian ethnicity are a likely, but not sufficient sources to explain the observed heterogeneity. In contrast, differences in DXA manufacturers and levels or correction for BMI do not seem to be an important source of heterogeneity.

Our study has limitations. We procured including all eligible studies to the best of our capacities but at least four studies were not able to be traced back. Sensitivity analyses considering such studies did not essentially change our results or conclusions. Variation in the definition of T2DM was present across studies with some combining self-reports and blood glucose tests, while others only used blood glucose tests. Studies which relied either on self-reports, population screening or which used register data will be subject to potential disease misclassification bias. Similarly, differences in mode of diagnosis can affect the prevalence of disease across studies and, hence, influence the power for detecting BMD differences. Disease duration can also be an important confounder, but uniform assessment for this co-variable was not possible across studies. Another drawback is that not all studies reported on or adjusted for covariates. Yet another potential source for heterogeneity that we could not control for are differences in glucose control and prevalence of diabetic complications. Nevertheless, the meta-regression done for BMD on the group of diabetic individuals across studies shows that in addition to BMI, HbA_1C_ levels also has a significant positive effect on BMD measured at any site.

Since May 2010 about 134 articles have been published on the topic of which we could identify two that would have met our inclusion criteria [55, 56]. These were studies based on Chinese populations showing opposite results with one concluding type 2 diabetics had higher BMD [55] while the other [56] concluded diabetics had lower BMD and higher risk of osteoporosis.

Mechanisms that might account for an association between T2DM and increasing BMD are plentiful and largely unclear. We discuss below from a clinical perspective the most important factors which can influence the relationship between T2DM and BMD.

### Obesity

Historically, overweight and hyperinsulinemia have been postulated as two important features of T2DM which are positively correlated with BMD. Yet, we saw that in a considerable number of the included studies the correction for BMI did not essentially modify the association. There are several complex pathways by which obesity may influence the relation between diabetes and BMD. Body fatness may have an impact on the accuracy of DXA-based BMD measures as demonstrated in obese diabetic patients [57]. Yet, such measurement error should be negligible considering that this phenomenon can either under or overestimate the values and have been shown to have low impact on the accuracy of the BMD measurement [58]. On the other hand, adipose tissue releases a wide variety of adipokines that have been implicated either directly or indirectly in the regulation of bone remodeling [59]. Plasma leptin concentrations have been shown to be higher in diabetic men than in healthy controls [60]. Leptin induces bone growth by stimulating osteoblast proliferation and differentiation in vitro [61–63] and it has also been shown to inhibit osteoclastogenesis through reducing RANK/RANK-ligand production and increasing osteoprotegerin [64, 65]. Other adipokines such as adiponectin and resistin are also expressed in osteoblasts and osteoclasts [66, 67]. The effects of these adipokines on bone metabolism remain largely ambiguous but differentiation from mesenchymal progenitor cells to osteo- or adipocytes may play a role [67–70]. Some reports indicate that circulating adiponectin [71] and resistin levels [72] are reduced in diabetes in line with a recent report demonstrating that higher adiponectin levels are associated with lower BMD [73].

### Hyperinsulinemia

Some of the reviewed studies indicated that insulin levels could mediate in part a positive association between T2DM and elevated BMD. Individuals with T2DM usually have an excess of insulin. Physiologically, insulin has an anabolic effect on bone due to its structural homology to IGF-1 by interacting with the IGF-1 receptor which is present on osteoblasts [74]. The IGF-1 signaling pathway is crucial for bone acquisition [75]: both human and mouse studies have demonstrated a significant positive association between IGF-1 and BMD [76, 77]. From this perspective it can be hypothesized that hyperinsulinemia could have a mitogenic effect on osteoblasts and their differentiation by stimulating the IGF-1 signaling pathway. Some indirect influences of insulin on bone formation could possibly be mediated by osteogenic factors such as amylin, osteoprotegerin, sex steroids and sex hormone-binding globulin (SHBG).

### Medication use

Thiazide use which is expected to be higher in diabetic individuals has also been associated with higher BMD at different skeletal sites [78, 79]. Similarly, statin use (also more prevalent in diabetics) is also associated with higher BMD [80, 81]. Nevertheless, several of the included studies controlled for medication use, and thus it is unlikely that this alone can explain the observed associations. On the other hand medication use can well be a source of the large heterogeneity observed in the meta-analysis.

### Paradoxically increased fracture risk

For many of the aforementioned mechanisms resulting in higher BMD it is rather difficult to fit their role in the paradoxically increased fracture risk. It has been well established that diabetic patients have impaired bone healing after fracture [82]. This probably indicates a compromise of both osteoclastic [82] and osteoblastic cell lineages [83], and possibly also on bone remodeling. Indeed, a recent study by Burghardt et al. [84] using high-resolution peripheral quantitative computed tomography (HR-pQCT) reported up to twice the cortical porosity observed in type 2 diabetes patients as compared to controls. The results of this pilot investigation provide a potential explanation for the inability of standard BMD measures to explain the elevated fracture incidence in patients with T2DM presenting with higher BMD levels. Specifically, the findings suggest that T2DM may be associated with an inefficient redistribution of bone mass and insufficient compensation for increased body mass, which may result in impaired bending strength. In addition, bone strength might be compromised through different mechanisms, such as increased production of non-enzymatic cross-links within collagen fibers, accumulation of advanced glycation end products [85], higher serum glucose levels that can negatively influence bone matrix properties [86] or indirectly as a consequence of sarcopenia [87]. Finally, patients with diabetes have increased fall risk, which can arise as a consequence of sarcopenia, retinopathy and/or neuropathy. Very recently, it has been shown how Type 2 diabetes underestimates the risk of fracture at a given BMD level [88], reason why the diabetic status is needed to be considered in risk fracture algorithms [89, 90].

## Conclusion

Our meta-analysis showed that diabetic individuals have higher BMD levels than non-diabetics independent of the skeletal site of measurement, gender, age, BMI or medication use. In addition, by applying a meta-regression we could establish that younger age, male gender, higher BMI and higher HbA_1c_ are positively associated with higher BMD levels in diabetic individuals. The potential mechanisms underlying these associations remain complex suggesting that several influential factors need to be considered while interpreting the association between T2DM and BMD. Large prospective studies are needed to establish the mechanisms underlying this association, and most importantly the relationship with fracture risk, the most adverse consequence of osteoporosis.
